# Structural Brain Changes Following Left Temporal Low-Frequency rTMS in Patients with Subjective Tinnitus

**DOI:** 10.1155/2014/132058

**Published:** 2014-06-03

**Authors:** Astrid Lehner, Berthold Langguth, Timm B. Poeppl, Rainer Rupprecht, Göran Hajak, Michael Landgrebe, Martin Schecklmann

**Affiliations:** ^1^Department of Psychiatry and Psychotherapy, University of Regensburg, Universitaetsstraße 84, 93053 Regensburg, Germany; ^2^Interdisciplinary Tinnitus Center, University of Regensburg, Universitaetsstraße 84, 93053 Regensburg, Germany; ^3^Department of Psychiatry, Psychosomatics, and Psychotherapy, Social Foundation Bamberg, Buger Straße 80, 96049 Bamberg, Germany; ^4^Department of Psychiatry, Psychosomatics and Psychotherapy, kbo-Lech-Mangfall-Klinik Agatharied, Norbert-Kerkel-Platz, 83734 Hausham/Obb., Germany

## Abstract

Repetitive transcranial magnetic stimulation (rTMS) of the temporal cortex has been used to treat patients with subjective tinnitus. While rTMS is known to induce morphological changes in healthy subjects, no study has investigated yet whether rTMS treatment induces grey matter (GM) changes in tinnitus patients as well, whether these changes are correlated with treatment success, and whether GM at baseline is a useful predictor for treatment outcome. Therefore, we examined magnetic resonance images of 77 tinnitus patients who were treated with rTMS of the left temporal cortex (10 days, 2000 stimuli/day, 1 Hz). At baseline and after the last treatment session high-resolution structural images of the brain were acquired and tinnitus severity was assessed. For a subgroup of 41 patients, additional brain scans were done after a follow-up period of 90 days. GM changes were analysed by means of voxel based morphometry. Transient GM decreases were detectable in several brain regions, especially in the insula and the inferior frontal cortex. These changes were not related to treatment outcome though. Baseline images correlated with change in tinnitus severity in the frontal cortex and the lingual gyrus, suggesting that GM at baseline might hold potential as a possible predictor for treatment outcome.

## 1. Introduction

Subjective tinnitus is the phantom perception of a sound in the absence of a corresponding objective sound source. With about 25% of adults in the US having experienced a ringing in the ears at least once [1], transient tinnitus is a common phenomenon. About 10–15% of the world population experience tinnitus in its chronic form [2]. While the majority of those 10–15% gets used to their tinnitus and is able to lead a normal life, in 1–3% of the general population tinnitus is experienced as extremely bothersome and debilitating. It can severely affect patients' everyday lives and is often accompanied by psychiatric comorbidities such as depressive syndromes or sleep disturbances [2, 3]. In order to improve existing treatment options and also to generate new treatment strategies for subjective tinnitus, it is mandatory to broaden knowledge on the neural mechanisms underlying the tinnitus percept.

More than 15 years ago it has been suggested [4, 5] and demonstrated [6] that tinnitus is related to alterations in the central nervous system. Furthermore, recent functional neuroimaging studies suggest [7–10] that, apart from the auditory cortex, widespread neural networks involving many different brain areas seem to be involved in the generation and maintenance of the phantom sounds as well as in the distress accompanied by the tinnitus percept [11, 12]. In addition to functional alterations within the brain, tinnitus has also been shown to be related to structural brain changes [13]. Studies using high-resolution magnetic resonance imaging (MRI) to compare the grey matter (GM) volume and cortical thickness of tinnitus patients with healthy control subjects have revealed alterations in the auditory cortex [14–16] and in subcortical parts of the central auditory pathway like the thalamus [17] and the right inferior colliculus [18]. Furthermore, alterations in grey matter volume and cortical thickness were also found in nonauditory brain locations [15, 17–21].

The knowledge that subjective tinnitus is associated with neural alterations suggests the therapeutic use of brain stimulation techniques such as repetitive transcranial magnetic stimulation (rTMS). The early finding that the auditory cortex is overly active in tinnitus patients [6] led to the idea of using low-frequency repetitive transcranial magnetic stimulation to modify the cortical hyperactivity in patients with phantom sounds [22]. Ever since then low-frequency rTMS has been investigated in an increasing number of studies (for a review, see [23]) showing that rTMS is effective with high interindividual variability. However, it is still difficult to identify predictors for treatment success [24]. The idea to use and improve rTMS as a treatment for tinnitus is further pursued though. To gain deeper insight into the mechanisms of rTMS treatment —and consequently to facilitate improvement of the therapeutic approach— the complementary use of both longitudinal neuroimaging and clinical assessment to measure rTMS effects in tinnitus patients is an important next step in tinnitus research [25]. The number of studies addressing this issue is limited so far. Some studies investigated the effect of low-frequency rTMS treatment on auditory evoked potentials and auditory steady state responses using electro- and magnetoencephalography (EEG/MEG) [26–28]. Two studies using single-photon emission computed tomography and functional magnetic resonance imaging (fMRI) found changes of neural activity in the temporal lobe, the right cingulate gyrus, and the uncus [26, 29]. While those studies have provided first insight in the functional alterations that are associated with low-frequency rTMS of the auditory cortex, there is no study which adds knowledge about structural alterations induced by rTMS treatment in tinnitus patients. Until now, only one study examined the effect of low-frequency rTMS over the left auditory cortex in healthy subjects using voxel based morphometry (VBM) [30]. The results suggest that five days of rTMS treatment leads to GM changes in the auditory cortex and the thalamus.

Based on all those results the current study was conducted with the following three research questions in mind: (1) is there a change in grey matter detectable in tinnitus patients after 10 sessions of rTMS treatment and after a follow-up period of 90 days? (2) Is there a relationship between the clinical outcome and the grey matter changes? (3) Can structural imaging be used as a predictor for outcome? To answer these questions we evaluated MRI scans of patients suffering from subjective tinnitus which were done routinely before and after low-frequency rTMS of the temporal cortex.

## 2. Materials and Methods

### 2.1. Subjects

Data from 77 patients (59 male, 18 female) with chronic tinnitus were included in the analyses. Patients with cardiac pacemakers, history of seizures, or any severe somatic, neurologic, or psychiatric disorder were excluded. The decision whether a patient was suffering from any severe somatic, neurologic, or psychiatric disorder was made by the physician, who decided about study inclusion based on the global clinical impression. One criterion for a severe somatic, neurologic, or psychiatric disorder was the need for an immediate therapeutic action for the treatment of this disorder. Another criterion was current hospitalization because of such disorder.

All patients were treated with rTMS and underwent MRI scanning before (baseline) and after (day 12) ten sessions of rTMS treatment. In a subgroup of 41 patients, an additional measurement was done after a follow-up period of three months (day 90). The total sample of 77 patients was therefore divided into two independent subgroups of one sample with two scans (*n* = 36) and one sample with three scans (*n* = 41). Demographical and clinical characteristics for both subgroups are shown in Table 1. Audiological data and a measure of hyperacusis were not available for all patients and could therefore not be included in the further analyses. Standardized pure tone audiometry data was available for 57 patients and revealed a mean hearing loss of 20.38 ± 12.14 [dB HL] (average of all thresholds measured bilaterally ranging from 125 Hz to 8 kHz). As a screening measure of hyperacusis, patients were asked whether “sounds cause pain or physical discomfort” [31]. Of the 61 patients who answered this question, 35 said “yes” and are therefore supposed to suffer from hyperacusis. Independent samples *t*-tests and Chi^2^-tests revealed no significant difference between the two independent subgroups concerning all variables reported in Table 1.

### 2.2. Repetitive Transcranial Magnetic Stimulation

rTMS treatment consisted of 10 treatment sessions on 10 consecutive working days. Patients were treated in the context of several clinical trials [32–34] or rTMS was done as compassionate use treatment between 2006 and 2009. Patients were stimulated over the left temporal cortex (1 Hz, 2000 stimuli/day, 110% resting motor threshold) which was localized either by using a standard procedure targeting the primary auditory cortex based on the 10–20 system [35, 36] or by using neuronavigation based on individual MRI/PET (positron emission tomography) images. In the latter cases, the area of increased activation within the primary auditory cortex was used as target area. Even if these two methods may have resulted in slightly different targets, the spatial difference is smaller than the spatial accuracy of rTMS treatment with the used figure-of-eight coil. For rTMS treatment, a Medtronic system with a figure-of-eight coil was used (90 mm outer diameter; Medtronic, Minneapolis, MN). The coil was held with a mechanical arm and placed over the left temporal cortex with the handle of the coil pointing upwards. During treatment, the patients were seated in a comfortable treatment chair. The resting motor threshold was measured once before the first treatment session and was defined as the minimal intensity at which at least four out of eight magnetically evoked potentials were ≥50 *μ*V in amplitude in the right abductor digiti minimi muscle [37]. All patients were treated at the Tinnitus Centre at the University of Regensburg, Germany, and provided written informed consent. The treatment protocol has been approved by the local ethics committee.

### 2.3. Clinical Assessment

For the assessment of demographical and clinical characteristics, the Tinnitus Sample Case History Questionnaire was used [38]. Tinnitus severity was assessed using the German version of the Tinnitus Questionnaire (TQ [39, 40]) and a numeric rating scale, which measured how loud the tinnitus was perceived (How strong or loud is your tinnitus at present?). This scale was rated from 0 (not loud at all) to 10 (extremely strong or loud). These measures were assessed before the first treatment session (baseline), after the last treatment session (day 12), and—for the subgroup of 41 patients with three images—after the follow-up period of three months (day 90).

### 2.4. Magnetic Resonance Imaging

A Siemens Sonata 1.5 Tesla whole body scanner (Siemens AG, Erlangen) with a standard 8-channel birdcage head coil was used to collect the anatomical images. For each subject and each time point, a high-resolution T1-weighted image was acquired using a magnetization-prepared-rapid-acquisition-gradient-echo- (MP-RAGE-) sequence (repetition time 1880 ms; echo time 3.42 ms; flip angle 15°; matrix size 256 × 256; number of slices 176; voxel size 1 × 1 × 1 mm³).

### 2.5. Data Processing and Statistical Analysis

For statistical analyses of the clinical data, PASW statistics 18 (SPSS Inc., Chicago, IL) was used. To test for changes in tinnitus severity, an analysis of variance (ANOVA) with the within-subjects factor time (baseline, day 12, day 90) was calculated for both the TQ and the loudness scale. In case of significant results, post hoc paired *t*-tests were done. For the group of 36 patients with only two assessments, paired *t*-tests were used to compare the TQ and loudness on baseline and day 12. All statistical tests were two-tailed. The level of significance was set at .05.

Processing and statistical analysis of the anatomical data were performed with the SPM8 software package (Statistical Parametric Mapping, Wellcome Trust Centre for Neuroimaging; http://www.fil.ion.ucl.ac.uk/spm). All anatomical images were visually examined for the presence of morphological abnormalities or artifacts. Preprocessing of the anatomical data was done using the standard procedure of the voxel based morphometry toolbox (VBM8 version 435, Structural Brain Mapping Group; http://dbm.neuro.uni-jena.de/vbm) for longitudinal data and involved intrasubject realignment, bias correction, segmentation, and normalization to the Montreal Neurological Institute (MNI) space. The default options of the standard procedure were not changed. As modulation is not necessary for longitudinal data, unmodulated images were used. Afterwards, a quality check was done using VBM8 before smoothing data with a Gaussian kernel of 8 mm full width at half maximum. Only grey matter images were used for further analyses. For the statistical analyses all voxels with a grey matter value below 0.1 were excluded to avoid edge effects around the border between grey and white matter. All analyses were done for the overall group of 77 patients (baseline and day 12 scans) as this group provided the highest statistical power. Additionally, all analyses were also done for the independent subgroups with two (*n* = 36) and three (*n* = 41) MRI scans. The following whole-brain analyses were performed.

(1) Grey matter images acquired at every time point were compared by estimating a flexible factorial model in SPM8 with the factors subject and time (baseline, day 12, and day 90).

(2) To test for correlations between the grey matter changes over time and changes in the clinical outcome parameters, difference images were calculated using the image calculator implemented in SPM8 (day 12-baseline; day 90-baseline) and correlated with the corresponding difference in the TQ and loudness scores.

(3) To find out whether grey matter images might be useful as a predictor for clinical outcome, baseline images were correlated with the difference in the TQ score (day 12-baseline). Please see Table 2 for an overview of all analyses done.

(4) For all analyses, the significance threshold was set to *P* < .001 (uncorrected) at voxel level and *P* < .05 (familywise error (FWE) corrected) at cluster level. Due to the nonisotropic smoothness of VBM data, correction for nonstationarity was applied. Anatomical Automatic Labeling (AAL; [41]) and the SPM Anatomy Toolbox [42] were used for anatomic labeling of significant clusters.

## 3. Results

### 3.1. Clinical Outcome

**Figure 1:**
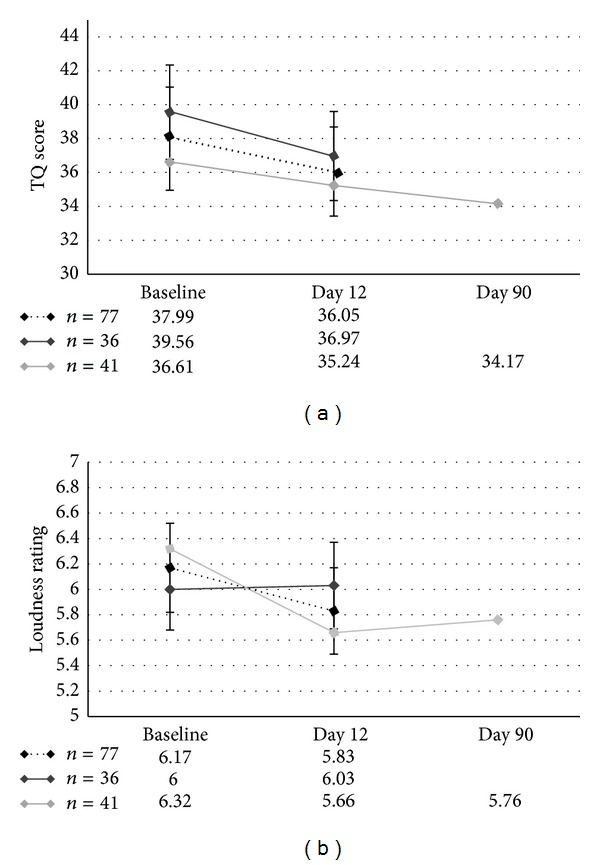
Line chart showing the time course of the TQ scores and the loudness ratings for both independent subgroups and the overall group.

The paired *t*-tests comparing the TQ and the loudness differences between baseline and day 12 in the overall group of 77 patients revealed a significant decrease in the TQ score (*t*(76) = 2.474, *P* = .016) and a marginally significant decrease in the loudness rating (*t*(76) = 1.745, *P* = .085). The paired *t*-tests comparing the TQ and the loudness differences between baseline and day 12 in the subgroup with only two scans (*n* = 36) revealed a significant decrease in the TQ score (*t*(35) = 2.292, *P* = .028) and no significant change in the loudness rating (*t*(35) = −0.099, *P* = .922). The ANOVA comparing the TQ scores of the subgroup with three scans (*n* = 41) revealed no significant effect of time (*F*(1.70, 67.82) = 1.743, *P* = .187). The ANOVA comparing the loudness scores of all three time points suggested a significant difference between at least two time points (*F*(2, 80) = 3.522, *P* = .034). Post hoc paired *t*-tests revealed a significant decrease from baseline to day 12 (*t*(40) = 2.529, *P* = .015) and a marginally significant decrease from baseline to day 90 (*t*(40) = 2.007, *P* = .052). There was no significant change from day 12 to day 90 (*t*(40) = −0.371, *P* = .713). See Figure 1 for a line chart showing the development of the TQ and loudness scores over time.

### 3.2. VBM

**Figure 2:**
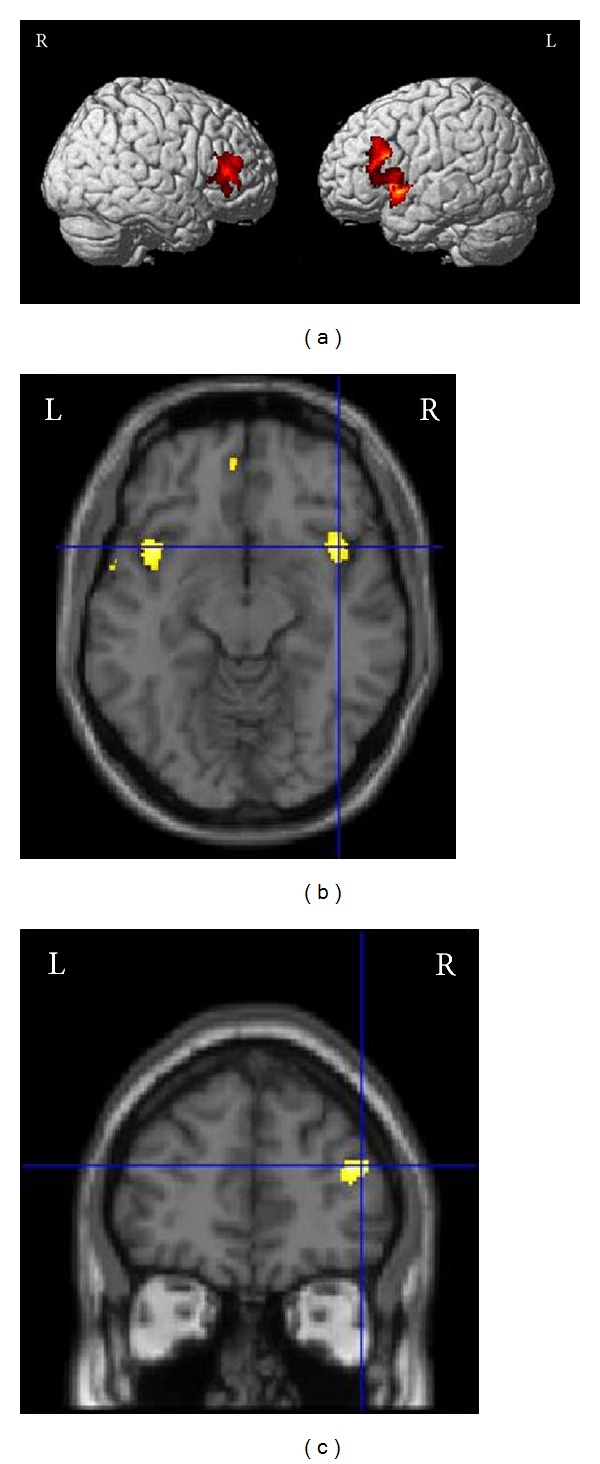
Grey matter decreases from baseline to day 12 in (a) the right and left inferior frontal gyrus and (b) the insula bilaterally. (c) Positive correlation of the TQ difference with the GM concentration at baseline in the right frontal gyrus.

**Figure 3:**
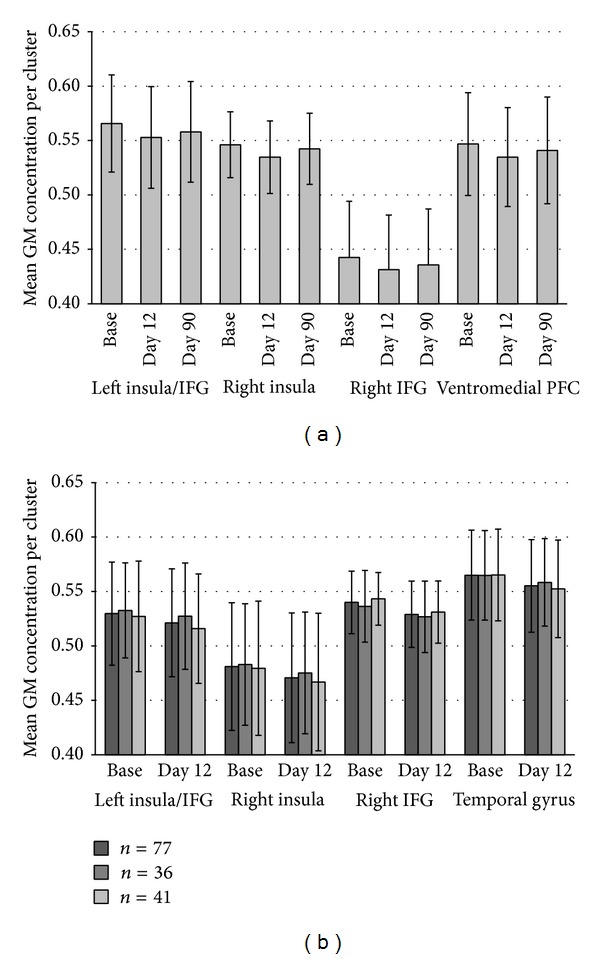
Mean grey matter concentration for each time point for the clusters with significant GM changes in (a) the subgroup of 41 patients and (b) the total group of 77 patients. For the clusters of (b) the mean GM concentration is also shown for the two independent subgroups.

(1) The flexible factorial models revealed significant grey matter concentration decreases from baseline to day 12 in the left and right insula as well as in the left and right inferior frontal gyrus (please see Figure 2 and Table 3 for MNI coordinates and statistical details). These GM changes were visible in both the *n* = 41 and the overall patient sample with 77 patients. It was not detected in the *n* = 36 sample though. If data of this group was analyzed with a more relaxed statistical threshold (*P* < .05 (uncorrected) at voxel level and *P* < .05 FWE corrected at cluster level), GM decreases were found in the right inferior frontal gyrus (*x* = 40, *y* = 39, and *z* = 19; *Z* = 3.07, *P* = .059). Please see Figure 3 for the mean GM concentration of the relevant clusters for all groups and all time points.

In addition, grey matter decreases were found in the left temporal pole and the left ventromedial prefrontal cortex. These GM changes were only visible in the *n* = 41 sample though. The contrast between baseline and day 12 in the overall patient sample (*n* = 77) additionally revealed decreased GM in the left inferior/medial temporal gyrus (Table 3). This was also visible in the *n* = 41 group (*x* = −62, *y* = −36, and *z* = −20; *Z* = 4.08, *P* = .016) if analyzed with a more relaxed statistical threshold (*P* < .001 (uncorrected) at voxel level and uncorrected at cluster level). In the *n* = 36 group, no significant GM decreases were visible. Overall, no grey matter increases from baseline to day 12 were visible in neither group. Neither grey matter increases nor decreases were found from baseline to day 90.

(2) The correlation analyses between the difference images and the difference in the TQ/loudness ratings revealed no significant results.

(3) The correlation analyses between the TQ difference and the baseline images revealed a positive correlation of the TQ with GM concentration in the left medial temporal pole and the right posterior cingulate cortex in the *n* = 36 group (Table 3). The correlations in the *n* = 41 group did not reach statistical significance. Furthermore, in the overall patient group, a positive correlation between the TQ difference and the baseline images was found in the left and right lingual gyrus. Additionally, a marginally significant positive correlation was detected in the right inferior/middle frontal gyrus. Using a more relaxed statistical threshold (*P* < .05 (uncorrected) at voxel level and *P* < .05 FWE corrected at cluster level), a marginally positive correlation in the lingual gyrus (*x* = −4, *y* = −91, and *z* = 13; *Z* = 3.78, *P* = .064) and in the inferior/middle frontal gyrus (*x* = 40, *y* = 44, and *z* = 21; *Z* = 3.34, *P* = .093) was also found in the *n* = 41 group.

## 4. Discussion

In order to improve rTMS treatment for patients suffering from subjective tinnitus, it is of particular importance to understand the neural alterations rTMS induces in tinnitus patients' brains in general and in treatment responders' brains in particular. The current study aimed at investigating the structural brain changes after rTMS treatment and the connection between these changes and clinical outcome. We examined grey matter alterations after ten sessions of low-frequency rTMS of the left temporal cortex. Besides the result that tinnitus severity and loudness were significantly reduced after rTMS treatment, the main findings of the present study were the following. (1) Transient GM decreases from baseline to day 12 were observed in several cortical areas. Neither GM increases nor GM changes from baseline to day 90 were detectable. (2) There was no correlation between GM changes and clinical outcome. (3) GM images at baseline correlated with treatment outcome suggesting that GM at baseline might be related to treatment response.

### 4.1. Grey Matter Changes from Baseline to Day 12

Bilateral GM decreases from baseline to day 12 were detectable in the insula and the inferior frontal gyrus (IFG). Those results were identical in the *n* = 41 group and the overall patient sample. On a more relaxed statistical threshold, the GM decreases in the right inferior frontal cortex were also visible in the *n* = 36 group. As it can be seen in Figure 3, this group also shows the tendency for GM decreases in both the right and left insula/frontal cortex. However, the difference is too small to reach statistical significance. Together with the anterior parts of the insula, the IFG is supposed to be a part of the ventral attention network (VAT), a mostly right-lateralized network responsible for a stimulus-driven “bottom-up” reorientation of attention to salient stimuli [43]. An altered connectivity between the VAT and the auditory and visual cortices in patients with bothersome tinnitus has recently been shown [44]. Furthermore, the insula has been reported to be part of a salience network [45], and both the IFG and the anterior insula are supposed to be involved in conflict processing [46]. If tinnitus is perceived as a permanent salient stimulus, it continuously attracts attention and conflicts with other salient stimuli. It is therefore not surprising that, as part of the VAT, alterations in the structure [15, 47] and function [10] of the IFG have been repeatedly reported in tinnitus research. While the insula is also a part of the VAT, it additionally plays an important role as part of a nonspecific distress network [11]. A relation between the insula and tinnitus distress has been consistently found in EEG studies [48, 49] and in studies examining structural brain alterations; decreased GM volume in the insula was reported in highly distressed patients [13] as well as a positive correlation between tinnitus distress and the cortical thickness in the anterior insula [19].

Notably, the GM decreases in the IFG and the insula seen in the current study were observed for the whole group independently of treatment outcome, indicating that these changes are rather related to the intervention than related to its clinical effect. The same is true for the remaining GM decreases observed. While GM alterations in the left temporal pole and the ventromedial prefrontal cortex were only visible in the small sample and are therefore not further discussed, the GM decrease in the inferior and middle temporal gyrus was only seen in the overall sample and—on a more relaxed statistical threshold—in the *n* = 41 sample. Again, the *n* = 36 sample showed the same tendency (see Figure 3) but not in a significant degree. Similar to the IFG and the insula, the medial temporal cortex has been previously reported to show functional alterations in tinnitus patients [10, 50]. However, GM changes in the medial temporal cortex might be rather linked to hearing loss than linked to tinnitus [14] and the same might be true for the inferior temporal cortex. Again, the morphological changes observed in the current study are not correlated with changes in the TQ or loudness scores. These results clearly suggest that rTMS leads to GM changes indeed but that these changes are an expression of “treatment” rather than an expression of “treatment outcome.” All in all, those results are to be seen as preliminary and replications are clearly needed as the GM decreases were only statistically significant in the overall sample and one subsample but not in the second, smaller subgroup of 36 patients.

Besides the GM decreases reported above, no grey matter increases were found from baseline to day 12—a finding which is not in line with the results of May et al. [30] who found GM increases in the left superior temporal area after 5 days of rTMS stimulation of the temporal cortex. The absence of such a GM increase in the current study is presumably not a problem of too little statistical power as it was found neither in the subsamples nor in the larger sample with 77 patients. One of the main differences between the current study and the study of May et al. is that the latter applied rTMS to healthy subjects while we used rTMS as a treatment for patients with subjective tinnitus. Maybe, tinnitus brains react differently to low-frequency magnetic stimulation in comparison to control subjects. Knowing that there are both structural and functional alterations in the tinnitus brain in comparison to healthy controls [8, 9] and knowing that the effect of 1 Hz-rTMS is state-dependent [26, 51] the different study outcomes might be reconcilable.

### 4.2. Grey Matter Changes from Baseline to Day 90

Interestingly enough, no GM decreases (nor increases) were seen from baseline to day 90 which suggests that the decreases seen on day 12 are temporary in nature. This observation is in line with the results of May et al. who also found that the changes induced by rTMS are transient [30]. It remains to be seen at which point in time the regression of the GM changes happens exactly. Whether the observed transient nature of the rTMS effect on GM may also reflect a transiency of clinical effects of rTMS treatment should be explored in further studies. Notably, previous long-term follow-up investigations in tinnitus patients have suggested long-lasting effects over periods of up to four years in the majority of rTMS responders [52, 53].

### 4.3. Grey Matter Changes and Clinical Outcome

Obviously, rTMS treatment of the temporal cortex leads to alterations in cortical regions known to be important for subjective tinnitus. These alterations do not seem to directly cause change in tinnitus distress though. As we investigated 77 patients, the lacking correlations do probably not arise from too little statistical power. Rather, it has to be considered that VBM might not be a method sensitive enough to capture neural changes that are related to the slight change of tinnitus distress or loudness which can be obtained using rTMS. This might be different for TMS treatment protocols with larger treatment effects and this might also be different for neuroimaging methods more sensitive to function rather than structure—such as fMRI or EEG. The only study investigating functional changes induced by rTMS using fMRI measurements could in fact not detect a relationship between changes in brain activity and clinical outcome [26]. However, with only six patients the study might have lacked the required power to detect such an effect.

Taken together, the key message is that rTMS treatment of tinnitus patients affects brain structures different to the stimulation site which points to the importance of interconnections between distant cortical areas. It is well-known that TMS effects are not limited to the stimulated area and that functional changes can also be seen in remote cortical brain areas [54, 55]. What is true for functional changes might also be right for structural changes. While May et al. [30] found GM increases in the stimulated area, they also reported the trend of GM increases in the temporal cortex contralateral to the stimulation site as well as in the thalamus bilaterally. Together with the results of the current study this emphasizes the importance of having in mind that magnetic stimulation of one cortical hotspot results in functional and presumably also structural alterations in a whole network of interconnected areas.

In summary, the bilateral alterations in the IFG and insulae after rTMS, although not seen on a significant level in the *n* = 36 group subgroup, further support the notion of functional connectivity between the left temporal cortex and the ventral attention network in tinnitus patients. Whereas rTMS induces transient alterations in these areas and also in the inferior and medial temporal cortex, these changes do not determine the clinical effects.

### 4.4. Baseline Grey Matter Images as Predictor for Treatment Outcome

Concerning the question whether grey matter images can serve as predictors for treatment response, the current results suggest that there are some cortical areas in which patients who will benefit from rTMS treatment have less GM at baseline than patients who will not benefit. In the right IFG and the lingual gyrus bilaterally, a positive correlation between GM at baseline and the TQ change was detected which means that an improvement in the TQ (implicated by negative values) is related to less GM at baseline. These results were seen in the overall patient group and in tendency also in the *n* = 41 group. Though a positive correlation was also found in the left medial temporal pole and the right posterior cingulate cortex, these results were only visible in the *n* = 36 sample and are therefore not further discussed. As mentioned above, the right IFG is part of the VAT and important for attention shifts to salient stimuli. The question arises however, what “reduced GM volume in the right IFG” actually means in terms of the function of the VAT. One could speculate that the VAT had been less sensitive to salient stimuli (e.g., the tinnitus) prior to rTMS treatment. As a consequence, a reduction of tinnitus severity might have been easier to accomplish in those patients. This is speculation though and—after replication—a challenging question for future research. The lingual gyrus has never been reported to play an important role for subjective tinnitus. However, functional and structural alterations in nearby occipital regions have been observed in tinnitus patients [14, 56], even if one of those studies suggests that GM decreases in occipital regions might be rather due to hearing loss than due to tinnitus [14]. Overall, these findings have to be considered as preliminary as the mentioned correlations reached statistical significance only in the overall patient group but not in the two independent subsamples. Therefore, replications are needed to confirm those results. Furthermore, there is some evidence that patients who benefitted from treatment once also benefit from a second treatment phase [57–59]. For that reason, future studies should also try to shed light on the question whether there are characteristics in the brain which predispose an individual to benefit from rTMS treatment in general while others do not.

### 4.5. Limitations

The current study has a number of limitations which should be considered in future studies. First, as just mentioned, hearing level was not available for all patients and could therefore not be integrated in the analyses. Although hearing loss is not supposed to be a predictor for response to rTMS treatment [24], previous studies have shown that hearing loss is an important confounder concerning GM changes in tinnitus patients [14, 60, 61]. To be able to thoroughly interpret research results, future work should try to include pure tone audiogram including high frequency audiogram [14, 60, 61] for all patients. Second, the lacking correlation between treatment outcome and GM changes might have been due to the small treatment effects. As already known from previous studies, the effect of rTMS treatment is small. Therefore, an even higher number of patients might have been necessary to ensure sufficient power for all analyses. The third and main limitation of the current study is the lack of a placebo condition. Without a patient group treated with sham stimulation we cannot definitely determine whether the observed GM changes were specific to rTMS treatment or unspecific effects. In the study of May et al. [30], healthy control subjects showed no GM changes after sham rTMS as opposed to subjects treated with active rTMS. This finding has not been replicated for tinnitus patients yet.

## 5. Conclusions

To the best of our knowledge, this is the first study to combine clinical assessment and longitudinal structural MRI scans to measure rTMS effects in tinnitus patients. The major result of the study is that ten days of low-frequency rTMS treatment of the temporal cortex leads to transient GM decreases in cortical regions different from the stimulated area. This highlights the importance of considering that the brain is organized in networks and that this organization highly influences the outcome of an intervention. Transient GM decreases were seen bilaterally in the insula, the IFG, and the left inferior/middle temporal gyrus, indicating functional connectivity between the stimulation site in the left temporal cortex and the ventral attention network in tinnitus patients. Although these cortical areas are known to be important in the generation and maintenance of tinnitus, the GM decreases were independent of treatment success. Thus, they were rather related to the TMS intervention per se and not to its clinical effect. However, treatment outcome correlated with GM at baseline indicating reduced GM in the right IFG and the lingual gyrus in patients benefiting from treatment. Thus, baseline GM images might hold potential to be further investigated as predictor for rTMS response in the future.

## Figures and Tables

**Table 1:** Demographical data and clinical characteristics for both independent subgroups.

	VBM data at baseline, day 12, and day 90 (*n* = 41)	VBM data at baseline and day 12 (*n* = 36)	Group comparison	
				*P* value
Gender	32 m (78%) 9 f (22%)	27 m (75%) 9 f (25%)	*χ* ^2^ (1.77) = 0.10	0.752
Age (years)	50.72 ± 13.37	50.79 ± 13.28	*T*(75) = −0.02	0.983
Tinnitus laterality	10% right 15% left 75% bilateral	14% right 14% left 72% bilateral	*χ* ^2^(2.77) = 0.32	0.853
Tinnitus duration (years)	8.97 ± 8.36	7.57 ± 6.74	*T*(75) = 0.80	0.427
TQ (baseline)	36.61 ± 17.78	39.56 ± 18.21	*T*(75) = −0.72	0.476
Loudness (baseline)	6.32 ± 2.04	6.00 ± 2.11	*T*(75) = 0.67	0.441
Mean hearing threshold [dB HL]	21.67 ± 11.49 (*N* = 29)	19.06 ± 12.85 (*N* = 28)	*T*(55) = 0.81	0.421
Hyperacusis	51% (*n* = 39)	68% (*n* = 22)	*χ* ^2^(2.61) = 2.31	0.316

TQ: Tinnitus Questionnaire.

Loudness: how STRONG or LOUD is tinnitus at present (0 not at all, 10 extremely strong or loud).

Mean hearing threshold: average of all thresholds measured bilaterally ranging from 125 Hz to 8 kHz).

**Table 2:** Overview over all VBM analyses.

Research question	Statistics		
	*n* = 41 (3 scans)	*n* = 36 (2 scans)	*n* = 77 (whole group 2 scans)
(1) Grey matter changes after rTMS?	Flexible factorial models with factors subject + time		
	Time points: baseline, day 12, day 90	Time points: baseline, day 12	Time points: baseline, day 12

(2) Correlation between grey matter changes and clinical outcome parameters?	Correlation of difference in the TQ/loudness rating with difference images		
	Time difference: day 12–baseline day 90–baseline	Time difference: day 12–baseline	Time difference: day 12–baseline

(3) Grey matter as predictor for treatment response?	Correlation of difference in the TQ with baseline images		

**Table 3:** Results of all VBM analyses.

Laterality	Anatomical region	Cluster size in voxels	MNI coordinates			Peak voxel *Z*-score	Cluster level *P* value
			*x*	*y*	*z*		
GM decrease from baseline to day 12 (*n* = 41)							
L	Temporal pole, insula, and inferior frontal gyrus	1121	−56	8	−18	4.93	<0.001
R	Insula (extending into temporal pole)	565	33	10	−18	4.79	0.001
R	Inferior frontal gyrus	475	51	33	12	4.49	0.009
L	Ventromedial prefrontal cortex	355	−4	52	−8	3.72	0.026

GM decrease from baseline to day 12 (*n* = 77)							
L	Inferior frontal gyrus, insula	1439	−46	12	−5	4.41	<0.001
R	Insula (extending into temporal pole)	684	42	16	−11	4.44	0.001
R	Inferior frontal gyrus	616	51	34	12	4.74	0.001
L	Inferior/medial temporal gyrus	558	−57	−42	−17	4.21	0.045

Positive correlation of TQ difference with baseline images (*n* = 36)							
L	Medial temporal pole	460	−32	6	−33	4.67	0.014
R	Posterior cingulate cortex	430	6	−45	31	4.19	0.036

Positive correlation of TQ difference with baseline images (*n* = 77)							
R + L	Lingual gyrus	534	4	−72	0	4.49	0.037
R	Inferior/middle frontal gyrus	413	52	30	19	3.86	0.089

FWE-corrected at cluster level *P* < 0.05.

L, left; R, right; MNI, Montreal Neurological Institute.
